# Effect of *Pseudomonas putida* on Growth and Anthocyanin Pigment in Two Poinsettia *(Euphorbia pulcherrima)* Cultivars

**DOI:** 10.1155/2014/810192

**Published:** 2014-07-03

**Authors:** Ramon Zulueta-Rodriguez, Miguel Victor Cordoba-Matson, Luis Guillermo Hernandez-Montiel, Bernardo Murillo-Amador, Edgar Rueda-Puente, Liliana Lara

**Affiliations:** ^1^Facultad de Ciencias Agricolas, Universidad Veracruzana, Circuito Universitario Gonzalo Aguirre Beltran S/N, Zona Universitaria, 91090 Xalapa, VER, Mexico; ^2^Centro de Investigaciones Biologicas del Noroeste, Instituto Politecnico Nacional 195, Colonia Playa Palo de Santa Rita, 23090 La Paz, BCS, Mexico; ^3^Universidad de Sonora, Boulevard Luis Encinas y Rosales, Colonia Centro, 23000 Hermosillo, SON, Mexico

## Abstract

*Pseudomonas putida* is plant growth promoting rhizobacteria (PGPR) that have the capacity to improve growth in plants. The purpose of this study was to determine growth and anthocyanin pigmentation of the bracts in two poinsettia* Euphorbia pulcherrima *cultivars (Prestige and Sonora Marble) using three strains of* P. putida*, as well as a mixture of the three (MIX). Comparison with the control group indicated for the most part that Prestige grew better than the Sonora Marble cultivars with the PGPR strains. Prestige with the MIX strain grew better compared to control for the number of cyathia (83 versus 70.4), volume of roots (45 versus 35 cm^3^), number of leaves (78 versus 58), and area of leaf (1,788 versus 1,331 cm^2^), except for the number of flowers (8.8 versus 11.6). To the naked eye, coloration of plants appeared identical in color compared to the control group. For all plants with* P. putida* strains, there was less anthocyanin pigment, but biomass was always greater with PGPR strains. Nevertheless, to the naked eye, the coloration of the plants appeared identical in color compared to the control group. This is the first study reporting the positive effects of* P. putida* rhizobacteria treatments on growth of poinsettia cultivars.

## 1. Introduction

The poinsettia* Euphorbia pulcherrima* of the family Euphorbiaceae is a member of the spurge family of plants which is made up of 300 genera and around 7,500 species; some have economic importance such as* Ricinus communis* (castor oil) or* Jatropha curcas* (biodiesel). The poinsettia is an ornamental plant of economic importance in Mexico, United States, and other countries. The majority of plants are sold in December with preferred colors being red, green, white, and purple colored plants. Red plants are particularly popular with worldwide sales of over $145 million [1].

Mexico is the fourth worldwide, producing an estimated 20 million plants per year with a value of 54 million USD, covering an area of 320 hectares. In cultivation of the poinsettia, it is important to produce plants with intense color foliage for consumer acceptance. The presence of anthocyanin determines the intensity or lack of red color in the bracts [2]. To achieve the color intensity, plant handling involves controlling photoperiod and nutrition. The application of chemical fertilizers can affect negatively nutrition and as a consequence the color in poinsettia plants. Hence, there is a growing interest in looking for options that reduce the use of chemicals to maintain plant health and reduce production costs. Among the options is the use of bacteria whose habitat is located in a zone surrounding the roots of the plants or rhizosphere. These bacteria, appropriately called rhizobacteria, are known as plant growth promoting rhizobacteria (PGPR) [3]. PGPR produce physiologically active substances that encourage seed germination, host growth acceleration, and increase crop yield, improving plant defenses against pathogens [4, 5]. Rhizobacteria have played an important role in moderating the excessive use of fertilizers and pesticides [6, 7].

PGPR within genera known to stimulate the growth of plants are* Azospirillum, Azotobacter, Bacillus, Enterobacter,* and* Pseudomonas*. The latter has been studied in recent years for its beneficial effects when used as organic fertilizer or as agents for biological control of pathogens [8]. One outstanding species is* Pseudomonas putida*, which is considered a metabolically versatile rhizobacterium ideal for agriculture applications.* P. putida* is known to quickly colonize the rhizosphere of plants thus outcompeting plant pathogens [9]. Despite the benefits found in various plants of agricultural interest [10], these rhizobacteria have not been evaluated thus far in poinsettia plants.

Among the few studies of rhizobacteria in ornamental plants is that of van Peer et al. [11] where they found that WCS417r strain of* P. fluorescens* protects carnation (*Dianthus caryophyllus* L.) plants against* Fusarium oxysporum*. Along the same lines of biological control, Shanmugam et al. [12] reported that* Bacillus atrophaeus* and* Burkholderia cepacia* substantially inhibit the development of* F. oxysporum* in gladiolus (*Gladiolus hortulanus* L. H. Bailey). In terms of improving growth, Sivakumar et al. [13] reported that inoculation of geranium (*Pelargonium graveolens*) with* P. fluorescens* enhances growth and biomass. Similarly, Padmadevi et al. [14] reported that the application of* Azospirillum* sp. and phosphate solubilizing bacteria (phosphobacteria) improves floral attributes (*Anthurium andreanum* Lind.) and that of Ambrosini et al. [15] where they reported that the genera* Burkholderia*,* Achromobacter*,* Chryseobacterium,* and* Azospirillum* had a stimulatory effect on plant growth of the sunflower (*Helianthus annuus* L.).

Poinsettia is a valuable ornamental plant with a short window of extraordinary commercial demand and sales during the month of December. To supply this demand peak, it is essential to have plants available within a short temporal space with their respective coloration. It is known in other plants that rhizobacteria microorganisms promote growth. Therefore, the purpose of this study was to determine the effect of three strains of* Pseudomonas putida* on growth and anthocyanin pigmentation in the bracts of two poinsettia cultivars.

## 2. Materials and Methods

### 2.1. Experimental Study Site

The experiment was conducted in a nursery located in La Estanzuela, Veracruz, Mexico, km. 5.5, Road Las Trancas-Coatepec, municipality of Emiliano Zapata (19°27′36′′N, 96°51′19′′W).

### 2.2. Bacterial Inoculum Preparation

We used three strains of* P. putida* labeled as FCA-8, FCA-56, and FCA-60 and prepared one MIX strain by mixing 20 mL of each of the three strains. These strains were originally isolated from* Citrus volkameriana* Tan. & Pasq. and provided by the Laboratorio de Quimica, Facultad de Ciencias Agricolas, Universidad Veracruzana, Xalapa, Veracruz, Mexico. All rhizobacteria were grown in King's B medium (glycerol 10 mL, protease-peptone 20 g, MgSO_4_ · 7H_2_O 1.5 g, K_2_HPO_4_ 1.5 g, agar 15 g, and sterile distilled water 1 L) at 28°C for 24 h. For all strains including MIX, the bacterial concentration was adjusted to 10^9^ cells mL^−1^ using a digital spectrometer Thermo Spectronic Genesys 20 (Thermo Electron Scientific Instruments Corp., Madison, WI) at a wavelength of 660 nanometers (nm) and absorbance of 1.

### 2.3. Vegetative Material Preparation

Cuttings were obtained of poinsettia Prestige and Sonora Marble cultivars from the International Poinsettia Nursery S.A. de C.V., located in Cuernavaca, Morelos, Mexico. The cuttings obtained for the two cultivars were 8 cm tall with 7 leaves. The substrate used consisted of pine sapwood (68%), tepezil (23%), and sand (9%) and was disinfected with 100 mL m^2^ of metam sodium (the third most commonly used agricultural pesticide in the USA). Pots of rigid polyethylene of 14 cm in diameter and with a height of 10.5 cm were filled with 500 g of the prepared substrate and one transplanted cutting was placed per container.

### 2.4. Experimental Inoculation

The roots of the cuttings of eight separate groups of seedlings of poinsettia Prestige and Sonora Marble cultivars (4 groups of each cultivar) were inoculated immediately at transplant with a single application of individual rhizobacteria strains FCA-8, FCA-56, FCA-60, and MIX by placing 2 mL · L^−1^ of the bacterial suspension directly to root before covering it with a soil mixture. A ninth group (control) only received fertilizer and water. All cuttings were fertilized with the recommended commercial dose every 15 days with 0.75 g L^−1^ of NO_3_ : P_2_O_5_ : K_2_O (15–05–25), 100 mg L^−1^ of calcium nitrate, 60 mg L^−1^ of micronutrients, and 0.05 mL L^−1^ of molybdenum. All cuttings were watered daily.

At 110 days after inoculation (DAI) the following was quantified: plant height (cm), stem diameter (mm), leaf area (cm^2^), number of cyathia, flowers, secondary stems, bracts and leaves, volume root (cm^3^), area of bracts (modified leaves red for Prestige and yellow for Sonora Marble cultivars, in units of cm^2^), fresh and dry biomass of bracts, leaves, stems, and roots. Ten replicates were used per treatment and the experiment was repeated twice.

### 2.5. Extraction of Anthocyanin

The anthocyanin pigments were extracted by macerating poinsettia bracts in water and filtering the mixture. Anthocyanin extraction was conducted with acidified methanol with 0.1 M HCl (85 : 15, v/v) [16, 17]. Once the anthocyanin sample was eluted, it was analyzed immediately so that the pigment would not degrade. This was carried out by diluting the sample and taking the spectral measurement.

### 2.6. Spectral Measurement of the Relative Anthocyanin Content in Bracts

The absorbance which is related to concentration of red pigment anthocyanin in poinsettia bracts was measured at *λ*_max⁡_ of 547 nm at pH 1 [18]. The reference blank contained only acidified methanol with 0.1 M HCl (85 : 15, v/v). For all spectral measurements, a spectrophotometer Thermo Spectronic Genesys 20 (Thermo Electron Scientific Instruments Corp., Madison, WI) was used. A total of 10 bracts were analyzed per treatment and the experiment was repeated twice.

### 2.7. Colonization Rhizobacteria

The total population of the* Pseudomonas* strains was determined at 110 DAI of cuttings by dilution technique on plate count following the methodology proposed by Holguin and Bashan [19]. A composite sample of roots of 1.5 g was collected following each treatment; it was macerated and dilutions were made in glass test tubes with 0.85% NaCl. The obtained dilutions were inoculated into a medium of cetrimide agar plates supplemented with 2 ppm of fluconazole which inhibits growth of filamentous fungi and incubated at 28°C for 72 h. Rhizobacterium population of each was expressed in colony forming units (CFU, 10^6^ g^−1^). There were three replicates per treatment and the experiment was repeated twice.

### 2.8. Statistical Analysis

The data were processed by one-way analysis of variance (ANOVA). We used the statistical package Statistica v. 10.0 for Windows (StatSoft) and post hoc Tukey's test (*P* < 0.05) was used for comparison of means.

## 3. Results and Discussion

The inoculation with different strains of* Pseudomonas putida* was found to have an influence on the growth and anthocyanin pigment contents of the bracts of poinsettia plants. The differences in growth parameters which included number of cyathia, number of flowers, volume of roots, number of leaves, area of leaf, fresh biomass of leaves and bracts, and area of bracts varied depending on the cultivar (Prestige and Sonora Marble) and strain of rhizobacteria in comparison to the control (not inoculated with rhizobacteria). Of the two cultivars, Prestige had a better response in terms of growth variables with the rhizobacteria.

For example, considering only Prestige (Table 1), the number of cyathia and flowers was greatest for FCA-60, root volume for MIX, number of leaves for FCA-56 and MIX, area of the leaf for FCA-8, FCA-56, and MIX, fresh biomass of leaves for FCA-60 and MIX and bracts for MIX, and area of bracts for FCA-8, FCA-60, and MIX. For Prestige, overall best strains for promoting growth and development compared to the control were in general F-60 and MIX. Strain FCA-60 promoted higher values for cyathia (36%), flowers (37%), and fresh biomass of leaves (22%) (Tables 1 and 2). All other growth parameters using F-60 with Prestige were not significantly different (*P* > 0.05). The inoculation with the three strains of* P. putida* (MIX) in Prestige increased the number of cyathia (17%), root volume (27%), number of leaves (35%), and area of leaf (34%) and had more fresh biomass of leaves (26%) and bracts (31%). For the MIX strain of Prestige only two growth parameters were not significantly different (*P* > 0.05); these were number of flowers and area of bracts.

For Sonora Marble, the growth parameters measured with the* P. putida* strains were not significantly different (*P* > 0.05) and for the majority of the strains were lower than the control. For Sonora Marble (Table 1), the number of cyathia was the greatest for control. For number of flowers and volume of root, the MIX and FCA-8 strains were the best, respectively. For number of leaves, the MIX and FCA-56 were the best. Sonora Marble did not show significant differences in bract area and fresh biomass (hence, data not shown), while Prestige did show differences of fresh biomass of leaves and bracts depending on the strain* P. putida* (Table 2). For Sonora Marble, the best strain for growth was the mixture of the three (MIX); individual strains generally did not improve growth parameters when compared to control (no rhizobacteria). The MIX strain in Sonora Marble increased the number of flowers (28%) and number of leaves (1.7%). However, the MIX strain also had lower values in other growth parameters such as for number of cyathia (8%), volume of roots (23%), and area of leaf (3.7%) in comparison with control.

The rhizobacteria apparently increased the capacity of absorption of nutrients and water in the roots of plants [20, 21] probably from the fertilization treatments. This has been shown to be the case in various cereals. In these studies, it has been found that by simultaneously applying nitrogen and phosphorus with rhizobacteria it was possible to improve growth, yield, and quality of grain [22]. Furthermore, the positive response of the plants due to the inoculation of the different strains of* P. putida* for both cultivars and in particular Prestige may be due to a hormonal effect of the rhizobacteria, whether it is being produced directly via an auxin and/or gibberellin [23]. These provide metabolizing signaling compounds that directly affect the regulation production of plant hormones [24, 25]. One of the main effects of this altered phytohormone production is a more elongated and nested root structure. Increased root volume allows for soil exploration improving water capture and nutrient assimilation [26]. However, it is important to note that the mechanism described above for* P. putida* is not the only possible explanation for plant growth. Another possibility could be related to its ability to better solubilize P, which can lead to a production of a wide variety of antimicrobial metabolites including enzymes, siderophores, volatile compounds, cyclic lipopeptides (CLPs), and antibiotics [27, 28].

**Figure 1:**
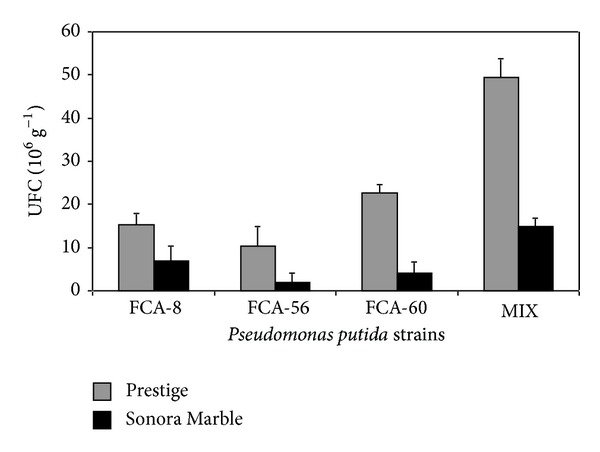
Population of* Pseudomonas putida* strains in the rhizosphere of poinsettia Prestige and Sonora Marble cultivars. Colony forming units (CFU) in plants with* P. putida* strain: FCA-8, FCA-56, FCA-60, and MIX (inoculated simultaneously with the three rhizobacteria) at 110 DAI. Data for controls of both Prestige and Sonora Marble cultivars are not shown since they were found to have 0 CFU.

Even though the strains of* P. putida* have a positive effect on plant growth in our experiment, Sonora Marble did not show a positive response in most of its other variables. This effect is possibly due to low* P. putida* colonization in the root of this cultivar (Figure 1), which was less compared to the other cultivars. For both Prestige and Sonora Marble cultivars, control groups without* P. putida* had 0 CFU colonization, indicating that there was no contamination with other bacteria (data not shown). One possibility for this behavior is that the Sonora Marble possibly exudates compounds in its roots which limit colonization of different strains of* P. putida* [29, 30]. In plants such as tomato, the colonization of the roots for rhizobacteria depends on a great variety of chemical compounds such as succinic acid, malic acid, L-aspartic, L-glutamic acid, L-isoleucine, L-leucine, and L-lysine [31]. It has been reported that Prestige exudates compounds in the root which are favorable for colonization [32]. Hence, in Prestige the establishment of its rhizosphere was probably aided by root exudates that favored* P. putida* colonization [33, 34]. Nevertheless, literature searches revealed no information about the type or amount of compounds in poinsettias exudates with rhizobacteria.

In regard to anthocyanin, it was found that in Prestige (red bracts) the absorbance was approximately two times higher than for Sonora Marble (yellow bracts), indicating that anthocyanin amount is lower in this cultivar (Table 3). Also found was that among the cultivar treatments the amount of anthocyanin varied very little compared to the control. This was corroborated by visual inspection since all treatments had the same characteristic color as the control.

## 4. Conclusion

As far as the authors are aware, this is the first report of inoculation of poinsettia (*Euphorbia pulcherrima*) plants with different strains of* Pseudomonas putida*, plant growth promoting rhizobacteria (PGPR). The highlight of this study is that the PGPR used in this study improved some growth parameters in two poinsettia cultivars (Prestige and Sonora Marble) and did not deteriorate poinsettia color. In terms of growth and developmental parameters, it was found that Prestige responded better to the individual strains of* P. putida* than Sonora Marble; possibly causes are root exudates that favored particular strains of PGPR more so in Prestige. In general, the data of this study also suggests that certain strains of* P. putida* can in particular cultivars of poinsettia improve growth parameters, but not in all. The long term perspective is to determine if* P. putida* will allow for reduction of fertilizer when growing poinsettia thus possibly allowing for improvement of production costs. It is also important to point out that this study, which identifies improvements in growth with* P. putida* rhizobacteria, is required before any detailed studies of mechanism(s) are undertaken. Studies of mechanism(s) are beyond the scope of the present study. However, having said this, it is important to point out that indeed future work will involve not only reduction of fertilizer, but also studies of the mechanism(s) of growth such as identifying growth hormones (such as IAA, gibberellic acid), determining P-solubilization and siderophore production.

## Figures and Tables

**Table 1:** Effects of *Pseudomonas putida* strains on growth promotion of poinsettia Prestige and Sonora Marble cultivars.

Strain *P. putida *	Number of cyathia		Number of flowers		Volume of roots (cm^3^)		Number of leaves		Area of leaf (cm^2^)	
	Prestige	Sonora Marble	Prestige	Sonora Marble	Prestige	Sonora Marble	Prestige	Sonora Marble	Prestige	Sonora Marble
Control	70.4^c^	97.2^a^	11.6^b^	35^b^	35.4^b^	16^b^	57.6^c^	93.8^b^	1,330.9^c^	1,265.3^c^
FCA-8	81.4^b^	62.2^d^	7.2^cd^	33.8^bc^	33.8^c^	26^a^	68^bc^	97.2^a^	1,737.3^ab^	1,627.1^a^
FCA-56	70.6^c^	87.4^b^	4.8^d^	32^c^	32^c^	17.6^b^	73^abc^	88.4^bc^	1,717.7^ab^	1,574.7^a^
FCA-60	95.8^a^	79.4^c^	16^a^	35^b^	35.8^b^	17.4^b^	67.8^bc^	83.8^bc^	1,481.6^c^	1,492.3^ab^
MIX	83^b^	90.6^b^	8.8^bc^	45^a^	45^a^	12.2^b^	78^a^	95.4^a^	1,788.1^a^	1,217^c^

Mean followed by the same letter in a column is not significantly different (Tukey, *P* > 0.05).

All plants were fertilized with the recommended commercial dose of 0.75 g L^−1^ of NO_3_ : P_2_O_5_ : K_2_O (15 : 05 : 25), calcium nitrate (100 mg L^−1^), micronutrients (60 mg L^−1^), and molybdenum (0.05 mL L^−1^).

MIX: plants simultaneously inoculated with strains FCA-8, FCA-56, and FCA-60 of *P. putida*.

**Table 2:** Effects of *Pseudomonas putida* strains on growth promotion of poinsettia Prestige cultivar.

Strain *P. putida *	Fresh biomass		Area of bracts (cm^2^)
	Leaves	Bracts	
Control	68.24^c^	40.2^b^	1,862.6^b^
FCA-8	79.25^abc^	48.1^ab^	2,440.3^a^
FCA-56	78.58^abc^	45.6^ab^	2,010^b^
FCA-60	83.32^ab^	51.7^ab^	2,173^ab^
MIX	86.34^a^	52.9^a^	2,125.8^ab^

Mean followed by the same letter in a column is not significantly different (Tukey, *P* > 0.05).

All plants were fertilized with the recommended commercial dose of 0.75 g L^−1^ of NO_3_ : P_2_O_5_ : K_2_O (15 : 05 : 25), calcium nitrate (100 mg L^−1^), micronutrients (60 mg L^−1^), and molybdenum (0.05 mL L^−1^).

MIX: plants simultaneously inoculated with strains FCA-8, FCA-56, and FCA-60 of *P. putida*.

**Table 3:** Effects of *Pseudomonas putida* strains on the anthocyanin pigment contents of the bracts of poinsettia plants.

Strain *P. putida *	Poinsettia cultivars	
	Prestige	Sonora Marble
Control	0.7427^a^	0.3876^a^
FCA-8	0.7230^ab^	0.3371^a^
FCA-56	0.6212^ab^	0.3150^a^
FCA-60	0.5900^b^	0.3150^a^
MIX	0.5651^b^	0.2692^a^

Mean followed by the same letter in a column is not significantly different (Tukey, *P* > 0.05).

All plants were fertilized with the recommended commercial dose of 0.75 g L^−1^ of NO_3_ : P_2_O_5_ : K_2_O (15 : 05 : 25), calcium nitrate (100 mg L^−1^), micronutrients (60 mg L^−1^), and molybdenum (0.05 mL L^−1^).

MIX: plants simultaneously inoculated with strains FCA-8, FCA-56, and FCA-60 of *P. putida*.
